# Large scale study of multiple-molecule queries

**DOI:** 10.1186/1758-2946-1-7

**Published:** 2009-06-04

**Authors:** Ramzi J Nasr, S Joshua Swamidass, Pierre F Baldi

**Affiliations:** 1 The Bren School of Information and Computer Science, Institute for Genomics and Bioinformatics, University of California, Irvine, CA 92697-3435, USA

## Abstract

**Background:**

In ligand-based screening, as well as in other chemoinformatics applications, one seeks to effectively search large repositories of molecules in order to retrieve molecules that are similar typically to a single molecule lead. However, in some case, multiple molecules from the same family are available to seed the query and search for other members of the same family.

Multiple-molecule query methods have been less studied than single-molecule query methods. Furthermore, the previous studies have relied on proprietary data and sometimes have not used proper cross-validation methods to assess the results. In contrast, here we develop and compare multiple-molecule query methods using several large publicly available data sets and background. We also create a framework based on a strict cross-validation protocol to allow unbiased benchmarking for direct comparison in future studies across several performance metrics.

**Results:**

Fourteen different multiple-molecule query methods were defined and benchmarked using: (1) 41 publicly available data sets of related molecules with similar biological activity; and (2) publicly available background data sets consisting of up to 175,000 molecules randomly extracted from the ChemDB database and other sources. Eight of the fourteen methods were parameter free, and six of them fit one or two free parameters to the data using a careful cross-validation protocol. All the methods were assessed and compared for their ability to retrieve members of the same family against the background data set by using several performance metrics including the Area Under the Accumulation Curve (AUAC), Area Under the Curve (AUC), F1-measure, and BEDROC metrics.

Consistent with the previous literature, the best parameter-free methods are the MAX-SIM and MIN-RANK methods, which score a molecule to a family by the maximum similarity, or minimum ranking, obtained across the family. One new parameterized method introduced in this study and two previously defined methods, the Exponential Tanimoto Discriminant (ETD), the Tanimoto Power Discriminant (TPD), and the Binary Kernel Discriminant (**BKD**), outperform most other methods but are more complex, requiring one or two parameters to be fit to the data.

**Conclusion:**

Fourteen methods for multiple-molecule querying of chemical databases, including novel methods, (ETD) and (TPD), are validated using publicly available data sets, standard cross-validation protocols, and established metrics. The best results are obtained with ETD, TPD, BKD, MAX-SIM, and MIN-RANK. These results can be replicated and compared with the results of future studies using data freely downloadable from http://cdb.ics.uci.edu/.

## Introduction

The rapid search of large repositories of molecules is a fundamental task of chemoinformatics. In a typical search, the molecules in a repository are ranked by their similarity to a single molecule query. If an appropriate similarity metric is employed, the molecules most similar to the query are most likely to exhibit physical, chemical, or biological properties similar to the query molecule. There is extensive body of literature on how to search based on this type of single-molecule query ([1-5] and references therein).

**Figure 1:**
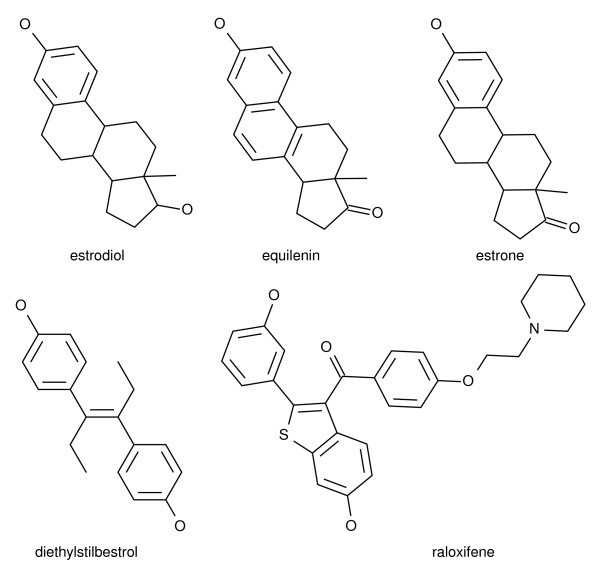
**A multiple-molecule query**. Examples of five estrogen receptor binding compounds that could be entered in a profile query.

However, in some chemoinformatic applications, several molecules of the same class are known. For example, given a set of molecules known to bind estrogen-receptor (Figure 1), one could search for additional estrogen-receptor binders. In these situations, searching the database with the whole group of query molecules may be more accurate than selecting a single-molecule to use as the query.

Multiple-molecule searches [4-8] have been less studied than single-molecules searches. Furthermore, the published studies of multiple-molecule searches suffer from critical weaknesses: reliance on proprietary data, poorly characterized background databases, and non-standardized performance metrics. Furthermore, some of these studies do not appropriately cross-validate their results.

Our study directly rectifies these deficiencies by using public data and better evaluation methodology. We both introduce novel multiple-molecule query methods and compare these methods with previously described multiple-molecule query methods using public activity data sets, a public background database, a strict cross-validation protocol, and reproducible evaluation methodology.

In what follows, many of the disparate methods from the literature are organized into a common framework.

## Molecular representations and similarity metrics

We begin by describing molecular representations and different metrics used to quantify the similarity between pairs of molecules. Multiple-molecule methods are often built on the pairwise-similarities between molecules.

There are many ways of measuring similarities between chemicals ([9-13] and references therein), but we focus on similarities measured between molecular fingerprints because they are the most commonly used in chemical search engines and the most studied.

### Molecular fingerprint notation

Molecular fingerprints are binary vectors where each component is a bit associated with the presence or absence of a particular feature (for example a functional group, path, tree, or some other graphical substructure) of labeled atoms and bonds present in the molecule. These vectors are typically long and sparse, on the order of 100, 000 or more bits long and with only hundreds of 1-bits for a given molecule.

Let  denote a molecule, and  = (*A*_*i*_) the corresponding fingerprint of length *N*, and *A*, the number of 1-bits in . Likewise, ℬ denotes another molecule,  = (*B*_*i*_) its corresponding fingerprint, and *B *the number of 1-bits in . Then *A *∪ *B *(resp. *A *∩ *B*) denotes the total number of 1-bits in  OR  (resp.  AND ). We can also define *A *⊕ *B *as the number of bits that are exclusively in either  or  but not both (the XOR), noting that this is exactly equivalent to (*A *∪ *B*) - (*A *∩ *B*).

For efficiency of space and time, fingerprints are often 'folded' to a much shorter length (*N *= 512 or *N *= 1024) using a lossy-compression algorithm. Each bit in the short, compressed fingerprint is indexed by *i *and each bit in the long, uncompressed fingerprint is indexed by *j*. The compression algorithm works by setting a 1-bit for each bit *i *in the short fingerprint if and only if there is at least a single 1-bit in one of the positions in the uncompressed, longer fingerprint which satisfies *j *mod *N *= *i*. This is the algorithm used by many chemoinformatic systems, including the Daylight system [3].

One can then compute similarity between either the longer, uncompressed fingerprints or the shorter, compressed fingerprints. Most similarity metrics are functions of *A*, *B*, (*A *∩ *B*), (*A *∪ *B*), and *N*, which can be directly computed from any pair of compressed or uncompressed fingerprints and then used to compute a similarity.

In choosing between compressed and uncompressed fingerprints, there is a tradeoff between efficiency and accuracy. The compressed fingerprints are faster and smaller but introduce systematic bias and random noise into any similarity computed from them. The folding compression algorithm artificially biases the similarities measured between folded-fingerprints to be higher than similarities computed between the corresponding unfolded-fingerprints. We have shown the bias introduced into some similarites by the folding-compression algorithm is both systematic and mathematically correctable [14]; using only information from the shorter, folded fingerprints, one can estimate the similarity between the unfolded fingerprints. This method improves on the tradeoff between efficiency and accuracy, yielding a system with high speed, low space, and high accuracy.

Where possible, we use corrected similarities. Occasionally, because of mathematical details of a particular similarity's formula, it is not possible to correct the similarity. The correction method assumes that *N *→ ∞, so when a similarity's formula includes *N *in a way which can not be algebraically removed, it cannot be corrected. For these cases, we compute similarity using compressed fingerprints, without the correction, and clearly note this in Table 1.

**Table 1:** List of method abbreviations.

Name	Equations	Parameters
**MIN-SIM**	18,1	0
**MAX-SIM**	17,1	0
**SUM-SIM**	16,1	0
**NUMDEN-SIM**	23,19,1	0
**MIN-RANK**	15,1	0
**MAX-RANK**	14,1	0
**SUM-RANK**	13,1	0
**BAYES**†	24	0
SUM-EH*†	16,11	2
SUM-ET*	16,12	2
SUM-TP*	16,9	1
**BKD**†	20,11	2
ETD*	20,12	2
TPD*	20,9	1

A list of abbreviated names for different multiple molecule queries. In the case of any methods with weight vectors, we use equally weighted convex combinations, in other words, w*i *= 1/|| where || is the number of query molecules. So, for example, the SUM-SIM becomes the average similarity. Methods introduced in this study for the first time are marked with a star (*). Methods using the compressed metrics are marked with a dagger (†); the rest use corrected metrics.

### Pairwise-molecular metrics

The multiple-molecule methods we will describe are generated by mathematically combining pairwise-molecular metrics. So, we must first define pairwise-molecular metrics before we define multiple molecule methods. We use the term 'metric' to include both similarities (denoted with an *S*) and dissimilarities (denoted with a *D*). Both can be used for searching by either selecting the most similar or the least dissimilar molecules from a query.

Several pairwise-molecule similarity metrics [15] have been introduced for molecular fingerprints, the most common one is the Tanimoto similarity. The Tanimoto similarity, also known as the Jaccard similarity, between two binary fingerprints is defined by the ratio of the number of bits set to 1 in both fingerprints to the total number of bits set to 1 in either fingerprint,(1)

The Tversky similarity is also commonly used, parameterized by 0 ≤ *α *and 0 ≤ *β*, which can be tuned to find approximate subsets and supersets of a query fingerprint,(2)

The Tanimoto similarity is identical to the Tversky similarity when *α *= *β *= 1.

Other metrics include the Cosine similarity,(3)

the Overlap similarity,(4)

and the Simple Matching similarity,(5)

One can also consider dissimilarities, which can often be generated by subtracting a similarity from a constant. For example, the Mismatch dissimilarity,(6)

is equivalent to *N *minus the Simple Matching similarity. In the case of binary vectors, this is also known as the Manhattan or Hamming distance.

Also, the Tanimoto dissimilarity,(7)

is equivalent to one minus the Tanimoto similarity.

These two dissimilarities are also mathematical distances, and each is associated with a specific similarity. The association between a similarity and a dissimilarity is important in some of the following definitions.

Additional metrics can be generated by considering monotone transformations of previously defined formulas. In the case of single-molecule queries, the rankings produced by monotone transformations of the same metric are exactly equivalent. However, in the case of multiple-molecule methods, where pairwise-metrics are mathematically combined to generate a composite score, monotone transformations applied to the pairwise-metric can substantially affect the final rankings.

The first, simple, non-linear transformation one can consider is raising a metric by a constant power,(8)

This equation is parameterized by the exponent, *α*, and is a monotonic transformation so long as *α *≠ 0.

Applying this to the Tanimoto similarity yields the Tanimoto-Power (TP) metric. This metric is defined as(9)

Other monotone transformations can generate additional metrics. For example, one can also consider the transformation(10)

where *S*(, ) is the similarity between *A *and ℬ, *D*(, ) = *C*_*S *_- *S*(, ) is the associated dissimilarity between  and ℬ, and *C*_*S *_is a constant fixed at the maximum possible value of the similarity *S*. The bandwidth parameter, *λ*, and the shape parameter, *k*, can be adjusted to fine tune this transformation to particular problems. Algebraically, this transformation is always monotone.

A special case of this more complex transform is used by some groups [6,8,16] where *S *is the Simple Matching similarity, therefore *C*_*S *_= *N*, and *D *is the Hamming distance, yielding(11)

We refer to this metric (Equation 11) as the Exponential Hamming (EH) metric, to emphasize it can be viewed as the Hamming distance (Equation 6) transformed by Equation 10.

We can also use this transformation using the Tanimoto similarity and the Tanimoto distance. In this case we have *C*_*S *_= 1 and(12)

This variation is termed the Exponential Tanimoto (ET) metric.

All three of these similarities, EH, ET, and TP are monotonic transformations of their underlying metrics (Tanimoto or Simple Matching). This means that the rankings for single molecule queries produced by ET and TP are exactly equivalent to those produced by Tanimoto similarity or distance. Likewise, the rankings produced by EH for single molecule queries are equivalent to the rankings produced by the Simple Matching similarity or the Hamming distance.

## Multiple-molecule methods

The multiple-molecule methods we describe are based on multiple-molecule similarities defined as  between the query molecules  and each molecule ℬ in the database. These similarities are the foundation of multiple-molecule search methods; they are used to rank the database and find the most relevant chemicals to a given query.

We can generate different multiple-molecule similarities by three general strategies: (1) aggregating the separately ranked results from single-molecule queries, (2) aggregating the individual pairwise similarity measures, and finally (3) aggregating the fingerprints into profiles and computing similarities between these profiles and single molecules.

### Aggregating ranks

In the first class of strategies, the idea is to aggregate the ranked results of single-molecule queries based on a pairwise-similarity, using each  separately. So rather than mathematically combining individual-pairwise similarities, we combine the ranked lists that these similarities generate. We must extend our notation to describe this class of approaches. While  denotes the similarity of molecule  and ℬ, we use  to denote the rank of molecule ℬ in the database  ordered by each molecule's pairwise similarity to .

In the top hits, molecules with the exact same similarity to the query occur frequently enough to require clear specification of 's treatment of molecules with tied score. In our formulation, ties are assigned cautious ranks that favor the inactive molecules. In other words, if active and inactive molecules are scored identically, the inactive molecules are assigned better ranks. Other ways of handling ties, which include the inverse of the former as well as assigning equal average ranks, yield very close results that are not significantly different.

By convention, lower ranks correspond to higher similarities, so we negate various aggregations of the single-molecule rankings to define new multiple-molecule methods. For example, we can define a new similarity as the weighted sum of the ranks(13)

the maximum (or worst) rank(14)

or the minimum (or best) rank(15)

Using a threshold of -*K *on the final formula (minimum of the ranks) is equivalent to pooling the top *K *hits, and removing redundant hits, from each single-molecule query.

### Aggregating similarities

In this class of strategies, the idea is to score the database by mathematically aggregating individual similarities between the database molecules and each of the query molecules. We define *S*(, ℬ) in terms of the individual similarities *S*(, ℬ) by taking the maximum, minimum, or weighted average. In the case of a weighted average with non-negative weights  we have(16)

where *S *is any of the similarities defined previously. Likewise, in the case where the similarity is defined by the maximum or the minimum we have(17)

or(18)

Within this class of strategies, it is also possible to consider measures that are obtained by combining the results of elementary pairwise comparisons between the molecules (e.g. intersections, unions) rather than the similarity measures themselves. In particular, one can derive a series of measures by simply aggregating the numerators and the denominators of the pairwise-similarity measures. In the case of the binary Tanimoto measure, we can define(19)

In some applications, in addition to scoring the database with information from active compounds, we can leverage information about compounds known to be inactive. Often, we can accurately infer that most database molecules are inactive and randomly select tens or hundreds from the database to use as a set of inactive molecules.

The Binary Kernel Discriminant (**BKD**) is an example of a method which uses information from inactive molecules. Aggregating the numerators and denominators of the Exponential-Hamming similarity, the **BKD **takes as arguments both a set of active molecules, , and a set of inactive molecules .(20)

This can be naturally varied by substituting different similarity metrics and aggregations, as has been done by at least one study [17]. The most important variation can be derived by substituting the corrected Exponential-Tanimoto (Equation 12) for the Exponential-Hamming, we call this method the Exponential Tanimoto Discriminant (**ETD**) or by substituting the Tanimoto-power (Equation 9) for the Exponential-Hamming, we call this method the Tanimoto Power Discriminant (**TPD**).

### Profile similarity

A third possible class of approaches to multiple-molecule queries is to aggregate fingerprints into a summary  = (*P*_*j*_) to represent the family  and then define similarity metrics between this profile and single molecules. The most common way of representing a family of molecules is with a consensus fingerprint where *P*_*j *_= min_*i*_*A*_*ij*_. So, *P*_*j *_= 1 only if all fingerprints in the family are one at the *j*th position. Similarity between the consensus fingerprint and a fingerprint  can be measured using any of the single-molecule similarity measures we have defined. For example, we could use Tanimoto similarity,(21)

Within this class of approaches, we can use a fingerprint profile to summarize the family in a more detailed manner than a consensus fingerprint. A fingerprint profile summarizes the information in a set of fingerprints, very much like a sequence profile or a position specific scoring matrix (PSSM) summarizes the information in a set of aligned sequences in bioinformatics. A fingerprint profile stores the relative frequencies that each bit is set to one in a set of fingerprints. If a given bit position is set to one in half the fingerprints, the corresponding component in the profile is set to 0.5. In addition, we can also assign different weights to each molecule or fingerprint in the family. In this most general case, . For proper scaling, it is desirable to use a convex combination with .

The similarity between the profile  and a fingerprint  can be measured using the MinMax (defined by Swamidass et. al. [12]) metric between scalar vectors,(22)

If the *A*_*i*_'s are binary and the combination is convex (), then we have the identity . Therefore, with convex linear combinations, we have the identity(23)

where *i *iterates over the fingerprints in the set of query molecules, and *j *iterates over the components of each fingerprint.

When we use a convex combination () on binary vectors, each component of  ranges from zero to one.  can be interpreted as a vector of probabilities. Once we think of the profile as a probabilistic model, we can apply priors and measure likelihood according to well-defined theory. Notably, similarity can be measured as the log likelihood of  according to ,(24)

This is equivalent to both the Naive-Bayes model used by [18,19] and to the Probability Scoring Matrix (PSM) commonly used in bioinformatics.

### Naming convention

In the following sections, for brevity and clarity, abbreviations are used to references each of the different of multiple molecule query methods. These names are tabulated in Table 1.

Some of these query methods presume the choice of a pairwise-similarity from which to derive the multiple-molecule similarity. Reported results use the Tanimoto similarity as this underlying pairewise-metric where possible, because it is the most commonly used and best performing pairwise-similarity. The exceptions to this decision are the **BKD **and **SUM-EH **methods, where the Hamming and Simple Matching metrics are used in order to replicate results from the literature.

Although not reported in this study, all experiments were replicated using three other pairwise-metrics known to perform well on chemical fingerprints: the Overlap, Mismatch/Euclidian, and Cosine metrics. Very rarely, the Cosine metric slightly outperforms the Tanimoto metric, but in all other cases the Tanimoto metric always outperforms other metrics. Most importantly, the particular single-molecule measure used did not affect the performance patterns of the multiple-molecule query methods. If a particular multiple-molecule method worked best for Tanimoto, it would also work best when other metrics were used.

### Data

other cases, they are known to elicit the same biological effect. Data sets include active molecules against HIV, different steroid receptors activists and antagonists, enzymes, and steroid families. See Table 2 enough to effectively evaluate methods in our largest data set, *suth-dhfr *with 722 chemicals. This background database is available for download at http://cdb.ics.uci.edu.

**Table 2:** Data set characteristics.

Name	Size	Reference
muv-chaperone	30	[25]
muv-gpcr-1	30	[25]
muv-gpcr-2	30	[25]
muv-gpcr-3	30	[25]
muv-kinase-1	30	[25]
muv-kinase-2	30	[25]
muv-kinase-3	30	[25]
muv-nr-1	30	[25]
muv-nr-2	30	[25]
muv-ppi-1	30	[25]
muv-ppi-2	30	[25]
muv-ppi-3	30	[25]
muv-protease-1	30	[25]
muv-protease-2	30	[25]
muv-protease-3	30	[25]
muv-rnase	30	[25]
muv-rtk	30	[25]
nci-hiv	415	[23]
stahl-cox2	125	[21]
stahl-estrogen	53	[21]
stahl-gelatinase	40	[21]
stahl-neuraminidase	17	[21]
stahl-p38-map-kinase	24	[21]
stahl-thrombin	67	[21]
suth-benzodiazepine	404	[22]
suth-dhfr	722	[22]
suth-estrogen	361	[22]
suth-steroid	28	[22]
wom-alr2	42	[20]
wom-androgen	36	[20]
wom-cdk2	152	[20]
wom-cox2	76	[20]
wom-d2	334	[20]
wom-egfr	74	[20]
wom-estrogen	64	[20]
wom-fxa	107	[20]
wom-hiv1rt	99	[20]
wom-impdh	49	[20]
wom-p38-map-kinase	59	[20]
wom-pde5	88	[20]
wom-ppar-gamma	27	[20]

Each data set is given an abbreviated name and is listed with the the number of molecules and its reference. In the case of the the NCI HIV screen (nci-hiv) we use only the 415 confirmed actives and discard the inactive and moderately-active compounds.

The multiple-molecule queries are benchmarked on a total of forty one data sets, divided into two groups. The first group of benchmarks use a background of negative examples randomly selected from the ChemDB. The second group of benchmarks uses data sets carefully derived from high throughput screening experiments specifically to more accurately assess query performance. This second group is expected to be more difficult than the first group.

First, we used twenty-four active data sets against a background database of molecules selected from the ChemDB. The data sets include thirteen WOMBAT data sets [20], six data sets from Stahl et al. [21], four data sets from Sutherland et al. [22] and the compounds with confirmed activity against HIV from the National Cancer Institute's (NCI) high throughput screen [23]. The background consists of 175, 000 randomly selected molecules. Based on the number of molecules in the active set, ||, Trunchon et. al. [24] derived a minimum size for the background database which ensures that there will be enough information for metrics to provide meaningful discrimination between compared methods. A background database size of 175, 000 corresponds to a maximum active set size of 860 actives: more than large enough to effectively evaluate methods in our largest data set, *suth-dhfr *with 722 chemicals. This background database is available for download at http://cdb.ics.uci.edu.

Second, we also used seventeen Maximum Unbiased Validation (MUV) data sets provided by Rohrer et al. [25] against their corresponding background databases. Each of these data sets include both 30 maximally dissimilar active compounds and a background set of 15,000 decoys similar to the actives in regards to low-dimension properties like solubility, volume, and surface area. These data sets were constructed specifically to avoid artificially high screening performance caused by using an inappropriate decoy data set. By selecting decoy data sets in this manner, it is assumed that the decoy chemicals are inactive even while there remains a small chance that they are active. In the case of the MUV data sets, the background decoys are selected from chemicals which have screened negatively and have, therefore, preliminary experimental evidence of inactivity. In contrast, by selecting the ChemDB background from untested chemicals, a stronger assumption that none of the untested chemicals are actives. There is a small chance that, for a small number of molecules, this assumption is not valid, and they are therefore incorrectly labeled as negative examples. This chance is sufficiently rare that it would not affect the performance metrics by an appreciable amount.

All of the forty-one data sets represent groups of diverse molecules with similar activity. In some cases, the molecules of each group are known to interact with the same protein. In other cases, they are known to elicit the same biological effect. Data sets include active molecules against HIV, different steroid receptors activists and antagonists, enzymes, and steroid families. See Table 2 for a full listing of the data sets.

For each data set we generate fingerprints with the same protocol using an in-house program written in Python. Fingerprints are associated with labelled paths of length up to 8 (i.e. 9 atoms and 8 bonds). In this case, the total number of observed labelled paths is about 150, 000. Compression is done using the lossy fingerprint folding algorithm. Results are reported for fingerprints of length *N *= 1024.

## Results and discussion

Different multiple molecule queries were assessed using a standardized protocol to calculate unbiased, quantitative measures of performance.

The fundamental, user-level goal of a multiple-molecule search is to identify additional molecules with the same biological activity as the query molecules. The performance at this specific task can be directly assessed. Using a standard leave-one-out (LOO) cross-validation protocol, we assign each molecule in the data set a LOO score, obtained by leaving it out from the classifier. So, for each molecule, its LOO score is defined as the score computed by ignoring any information known about just this molecule's class.

From these LOO scores, each method's performance, its ability to separate positive from negative examples, can be quantified using different performance metrics. Many performance measures have been used to quantify the performance of different query methods; Truchon et. al. [24] (and references therein) provide a useful review of both these measures and their pitfalls. More importantly, they derive a better measure, Boltzmann-Enhanced Discrimination of Receiver Operating Characteristic (BEDROC), which is designed specifically to evaluate virtual screening methods such as those we consider here. In this study, we evaluated different methods using both BEDROC, the Area Under the ROC Curve (AUC), Area Under the Accumulation Curve (AUAC), and the F1 score. The F1 score is a commonly used metric within the information retrieval literature,(25)

where *T P*, *F P*, and *F N *are the number of true positives, false positives, and false negatives at a particular threshold. For this study, the threshold measure which optimized F1 was chosen. The definitions of the other metrics can be found in the references.

All of the performance metrics require a fully ordered ranking to be computed, and cannot, therefore, handle ties appropriately. Of note, some methods output the same score for several chemicals, yielding ranked lists with ties. In these situations, the performance is computed by averaging twenty ranked lists sampled so as to randomly resolve ties differently each time. The performance across these samples is averaged and reported.

In total, fourteen similarity methods are evaluated with four performance metrics on 41 data sets resulting in 2296 measurements. For brevity, only summarized BEDROC, F1 and AUC performances are discussed. The complete results, including the AUAC performance, are included as additional files. [See Additional files 1, 2, 3].

In addition to reporting the performance of each method, the Receiver Operating Characteristic (ROC) curves and pROC curves [26] are used to display performance levels graphically. We use these established information-retrieval measures, using repeatable methodology, so our results can be directly compared to future studies.

For directly comparing different methods across the data sets, we compute a p-value using Welch's *t*-test. Sets of measurements corresponding to different similarity method are treated as populations in comparison. Over all data sets, this p-value reflects the confidence a given method is out-performing another according to a particular performance measure. Table 3 compares average results of the similarity methods across the 24 data sets with the ChemDB background. Similarly, Table 4 shows those results for the 17 MUV data sets. The best result per performance metric is shown in **bold **and other results that are statistically indistinguishable from the best (p-value > 0.05) are *italicized*. In Table 3, **ETD **outperforms the other methods, in many cases not by a significant margin. The MUV data sets results in Table 4 show again that **ETD **has the highest performances, depending on the metric used. BEDROC, however, is the preferred metric by which **ETD **outperforms the other methods.

**Table 3:** Mean performance of similarity methods.

Method	AUC	F1	BEDROC
**MIN-RANK**	*0.981265 *± 0.004540	*0.480749 *± 0.019308	*0.915781 *± 0.012475

**MAX-RANK**	0.633951 ± 0.038076	0.030289 ± 0.009587	0.209204 ± 0.049180

**SUM-RANK**	0.840620 ± 0.032520	0.128860 ± 0.032596	0.490227 ± 0.066911

**MAX-SIM**	0.973312 ± 0.005642	*0.484504 *± 0.022397	*0.893180 *± 0.015592

**MIN-SIM**	0.717104 ± 0.034874	0.053943 ± 0.015827	0.284041 ± 0.058423

**SUM-SIM**	0.914782 ± 0.018230	0.341373 ± 0.032341	0.719190 ± 0.041269

**NUMDEN-SIM**	0.907632 ± 0.019609	0.327810 ± 0.037178	0.696596 ± 0.044681

**BAYES**	0.910909 ± 0.017386	0.149837 ± 0.033785	0.581197 ± 0.050633

**BKD**	*0.980763 *± 0.004517	*0.501197 *± 0.019569	*0.890840 *± 0.017745

**ETD**	**0.987087 **± 0.002653	**0.508081 **± 0.020886	**0.922371 **± 0.011330

**TPD**	*0.986616 *± 0.002649	0.451587 ± 0.025795	*0.906017 *± 0.014098

**SUM-EH**	0.935054 ± 0.010774	0.296279 ± 0.032160	0.699456 ± 0.037583

**SUM-ET**	0.974831 ± 0.005798	*0.491106 *± 0.022314	*0.897401 *± 0.015760

**SUM-TP**	0.974963 ± 0.005751	*0.490653 *± 0.022311	*0.897621 *± 0.015771

The mean performance of similarity methods across the 24 data sets with the ChemDB background. A confidence interval is provided with each measurement. The best performance in each column is listed in bold face, and all performances statistically indistinguishable (with a *t*-test yielding a p-value > 0.05) are listed in *italics*.

**Table 4:** Mean performance of similarity methods across MUV data sets.

Method	AUC	F1	BEDROC
**MIN-RANK**	*0.731133 *± 0.030578	*0.149965 *± 0.023025	*0.345171 *± 0.042642

**MAX-RANK**	0.509469 ± 0.020590	0.017739 ± 0.004382	0.061569 ± 0.010419

**SUM-RANK**	0.598784 ± 0.030562	0.021604 ± 0.005490	0.104799 ± 0.022261

**MAX-SIM**	0.714848 ± 0.028352	*0.156955 *± 0.025644	*0.312150 *± 0.041033

**MIN-SIM**	0.533202 ± 0.025204	0.020921 ± 0.004781	0.070374 ± 0.008572

**SUM-SIM**	0.617073 ± 0.034809	0.052993 ± 0.021674	0.153437 ± 0.040308

**NUMDEN-SIM**	0.644467 ± 0.032684	0.061232 ± 0.022654	0.177026 ± 0.040264

**BAYES**	0.642907 ± 0.031377	0.041962 ± 0.011625	0.176723 ± 0.039162

**BKD**	*0.784118 *± 0.025509	*0.145667 *± 0.027758	*0.354250 *± 0.044227

**ETD**	**0.785944 **± 0.025833	*0.141997 *± 0.026715	**0.356733 **± 0.043394

**TPD**	*0.775774 *± 0.025289	*0.152530 *± 0.023448	*0.352975 *± 0.041960

**SUM-EH**	0.679485 ± 0.030601	*0.100943 *± 0.025459	0.230528 ± 0.046206

**SUM-ET**	*0.733893 *± 0.027988	*0.155680 *± 0.026528	*0.324622 *± 0.042935

**SUM-TP**	*0.729849 *± 0.028113	**0.157239 **± 0.026507	*0.323068 *± 0.042618

The mean performance of similarity methods across the 17 MUV data sets with their corresponding backgrounds. A confidence interval is provided with each measurement. The best performance in each column is listed in bold face, and all performances statistically indistinguishable (with a *t*-test yielding a p-value > 0.05) are listed in *italics*.

Different data sets can yield quite different performances; the performance variability between data sets is greater than the variability between the best methods. The average difference between a method from the best performing method, therefore, is a more statistically powerful assessment of performance. Tables 5 and 6 show the difference, averaged across the ChemDB background data sets and the MUV data sets respectively, between each method and the best performing method. Similar to Table 3 and Table 4, values that are statistically indistinguishable from that of the best method are *italicized*. The p-value in this case is derived from a paired *t*-test on the performance measurements.

**Table 5:** Mean difference in performance of similarity methods.

Method	AUC	F1	BEDROC
**MIN-RANK**	0.005821 ± 0.002344	0.027332 ± 0.006678	0.006590 ± 0.003236

**MAX-RANK**	0.353136 ± 0.036644	0.477792 ± 0.020875	0.713166 ± 0.043907

**SUM-RANK**	0.146467 ± 0.030849	0.379221 ± 0.028884	0.432144 ± 0.059736

**MAX-SIM**	0.013775 ± 0.003534	0.023577 ± 0.005890	0.029191 ± 0.007341

**MIN-SIM**	0.269982 ± 0.033128	0.454138 ± 0.023192	0.638330 ± 0.052591

**SUM-SIM**	0.072305 ± 0.016279	0.166707 ± 0.025321	0.203181 ± 0.032996

**NUMDEN-SIM**	0.079455 ± 0.017748	0.180270 ± 0.031082	0.225774 ± 0.036813

**BAYES**	0.076178 ± 0.016079	0.358244 ± 0.032005	0.341173 ± 0.045043

**BKD**	0.006324 ± 0.002360	*0.006884 *± 0.008400	0.031531 ± 0.008401

**ETD**	**---**	**---**	**---**

**TPD**	*0.000471 *± 0.000443	0.056493 ± 0.010274	0.016353 ± 0.004338

**SUM-EH**	0.052032 ± 0.009065	0.211802 ± 0.026177	0.222915 ± 0.031105

**SUM-ET**	0.012256 ± 0.003648	0.016975 ± 0.006151	0.024970 ± 0.007082

**SUM-TP**	0.012124 ± 0.003584	0.017428 ± 0.004657	0.024749 ± 0.007079

The mean difference in performance of similarity methods from that of the best method across the 24 data sets with the ChemDB background. A confidence interval is provided with each measurement. All performances statistically indistinguishable (with a paired *t*-test yielding a p-value > 0.05) are listed in *italics*.

**Table 6:** Mean difference in performance of similarity methods across MUV data sets.

Method	AUC	F1	BEDROC
**MIN-RANK**	0.054811 ± 0.007343	*0.007274 *± 0.009481	*0.011562 *± 0.013673

**MAX-RANK**	0.276475 ± 0.021613	0.139500 ± 0.023056	0.295164 ± 0.036878

**SUM-RANK**	0.187161 ± 0.021317	0.135635 ± 0.020176	0.251934 ± 0.029684

**MAX-SIM**	0.071096 ± 0.008692	*0.000284 *± 0.005657	0.044583 ± 0.013589

**MIN-SIM**	0.252742 ± 0.020037	0.136318 ± 0.021634	0.286359 ± 0.031277

**SUM-SIM**	0.168871 ± 0.018754	0.104246 ± 0.014075	0.203296 ± 0.017032

**NUMDEN-SIM**	0.141477 ± 0.019134	0.096007 ± 0.014050	0.179707 ± 0.018725

**BAYES**	0.143037 ± 0.016147	0.115277 ± 0.014736	0.180010 ± 0.012881

**BKD**	*0.001826 *± 0.001822	*0.011572 *± 0.009888	*0.002483 *± 0.005203

**ETD**	**---**	*0.015242 *± 0.010510	**---**

**TPD**	0.010170 ± 0.003688	*0.004709 *± 0.007578	*0.003758 *± 0.007367

**SUM-EH**	0.106459 ± 0.013059	0.056296 ± 0.007868	0.126204 ± 0.012854

**SUM-ET**	0.052051 ± 0.010111	*0.001559 *± 0.004737	0.032111 ± 0.012709

**SUM-TP**	0.056095 ± 0.009880	**---**	0.033664 ± 0.012780

The mean difference in performance of similarity methods from that of the best method across the 17 MUV data sets. A confidence interval is provided with each measurement. All performances statistically indistinguishable (with a paired *t*-test yielding a p-value > 0.05) are listed in *italics*.

In the next subsections, we explore graphical comparisons between methods within the same class of approaches. All ROCs and pROCs are leave-one-out (LOO) cross-validated and aggregated over all data sets with the ChemDB background. The plots visually illustrate qualitative comparisons of each method.

### Aggregating ranks

**Figure 2:**
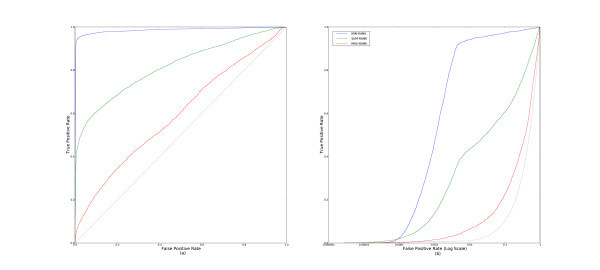
**ROC and pROC curves of multiple-molecule query methods that aggregate ranks**. This figure compares the parameter-free methods, **MIN-RANK**, **MAX-RANK**, and **SUM-RANK **with (a) complete ROC curves, and (b) pROC curves.

First, we examine the different methods based on rank aggregation. Figure 2 shows the cross-validated (a) ROC and (b) pROC curves derived by aggregating over all data sets. We can clearly see that **MIN-RANK **performs better than **MAX-RANK **and **SUM-RANK **by a substantial margin.

### Aggregating similarities (parameter-free)

We next examine the different parameter-free methods of similarity aggregating; the methods which require fitting parameters from the data are not discussed here, but in a later section.

**Figure 3:**
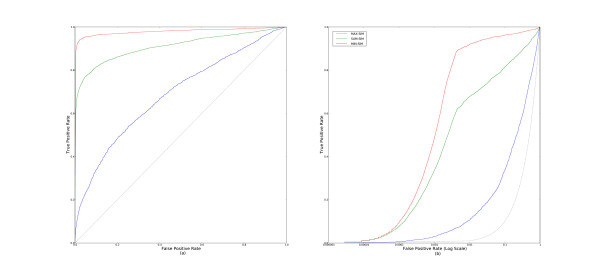
**ROC and pROC curves of multiple-molecule query methods that aggregate similarities with no learned parameters**. This figure compares the parameter-free methods, **MAX-SIM**, **MIN-SIM**, and **SUM-SIM **with (a) complete ROC curves, and (b) pROC curves.

By convention, lower ranks correspond with higher scores. So, if **MIN-RANK **performs best, we would also expect **MAX-SIM **to perform better than other methods of aggregating similarities. This is exactly what we observe. Figure 3 shows the cross-validated and aggregated (a) ROC and (b) pROC curves for the parameter-free similarity aggregating methods.

### Profile similarity and aggregating numerators and denominators

For Tanimoto similarity, MinMax similarity computed between a profile vector and a database fingerprint (Equation 22) exactly corresponds with separately aggregating the numerators and denominators (**NUMDEN-SIM**, Equation 19). So, in the case of binary fingerprints, the only distinct profile similarity method we describe is **BAYES **method (Equation 24).

**Figure 4:**
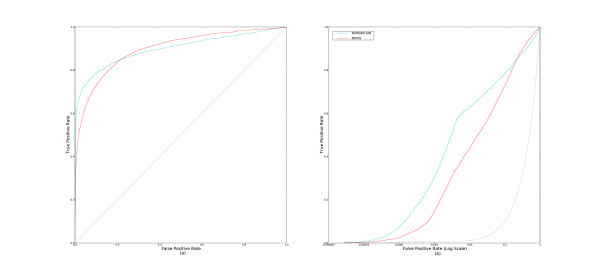
**ROC and pROC curves of BAYES and NUMDEN-SIM**. This figure shows the (a) ROC and (b) pROC curves for **BAYES **with **NUMDEN-SIM **included for comparison.

The **BAYES **method is used extensively by [18,19,27]. However, in our experiments it performs surprisingly poorly. Figure 4 shows the ROC and pROC curves for **BAYES **and **NUMDEN-SIM **for comparison. **NUMDEN-SIM **clearly outperforms **BAYES**

### Aggregating similarities (with learned parameters)

In this section, we consider methods which learn one or two parameters from the data. These methods were easily trained using a standard leave-one-out procedure to select the best parameterization from an exhaustive parameter sweep. Although one can imagine more efficient means of training these models, this is consistent with the literature. In turn, we first evaluate **SUM-ET**, **SUM-EH**, and **SUM-TP**, which learn using only an active set of molecules, and then **ETD**, **BKD**, and **TPD**, which use additional information from inferred inactive molecules randomly chosen from the background database.

**Figure 5:**
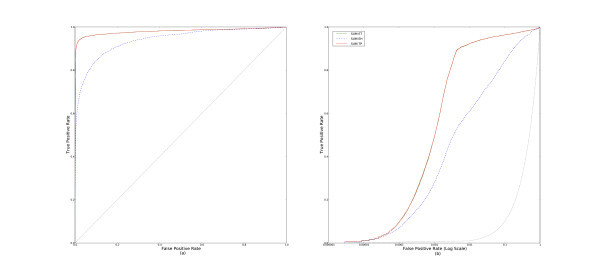
**ROC and pROC curves of two-parameter multiple-molecule query methods (only active sets)**. This figure compares the learned methods that use only an active set of molecules, **SUM-TP**, **SUM-ET**, and **SUM-EH **with (a) complete ROC curves, and (b) pROC curves.

**Figure 6:**
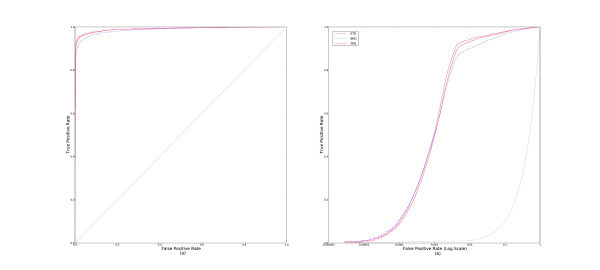
**ROC and pROC curves of two-parameter multiple-molecule query methods**. This figure compares the learned methods, **TPD**, **ETD**, and **BKD **with (a) complete ROC curves, and (b) pROC curves.

Results of **SUM-ET**, **SUM-TP**, and **SUM-EH **are shown in Figure 5. The first two methods perform at almost the same level, and outperform **SUM-EH**. The three methods which use additional information from inferred inactive compounds, **ETD**, **BKD**, and **TPD**, are all high performers as well. Figure 6 displays the aggregated ROC and pROC curves of the three methods. We see that although the curves are very close in Figure 5 that **ETD **slightly outperforms the other two methods.

### Best methods

**Figure 7:**
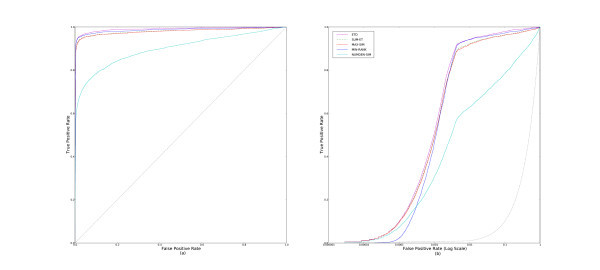
**ROC and pROC curves of the best multiple-molecule query methods**. This figure compares both the (a) complete ROC curves, and (b) the pROC curves of some of the the best performing methods: **ETD**, **SUM-ET**, **MAX-SIM**, **MIN-RANK**, and **NUMDEN-SIM**.

In this subsection, we examine the best methods from the above subsections. Only one of the best methods from each of the classes is chosen as a representative. Figure 7 displays the ROC and pROC curves of **ETD**, **SUM-ET**, **MAX-SIM**, **MIN-RANK**, and **NUMDEN-SIM**.

From the figure, we observe that **ETD **consistently outperforms **SUM-ET **by a small margin. There is little gain in performance from the information the inferred inactive compounds are adding. **MINRANK **consistently outperforms **MAX-SIM **and outperforms **ETD **in the early section of the curves (Figure 7(b)). **NUMDEN-SIM**'s performance falls behind the other methods'.

In most cases, we expect **MAX-SIM **and **MIN-RANK **to be of the most use because of their high performance and simple implementation. However, the stronger performance of the methods with learned parameter(s) suggests a small performance gain can be realized from the added effort of tuning a query to each data set.

## Conclusion

Here we have described and evaluated a large number of both novel and established methods of performing multiple-molecule queries using publicly available data, cross-validation protocol, and the best performance metrics.

Our benchmarks indicate that **MAX-SIM **and **MIN-RANK**, are the best performing methods which do not require parameters to be learned from the data. This is consistent with previous studies. Furthermore, the **ETD **is the best parameterized multiple-molecule querying method. Although the higher results are not always by a significant margin over **TPD **and **BKD**, it is consistent across our data sets and, to some extent, theoretically justifiable from previous work, but it is the first study of its kind on publicly available data and the first study to compare methods using bias-corrected similarity. All performance metrics were computed by evaluating the retrieval of chemicals with similar biological activity from a diverse random background and a background of decoys.

**BAYES **did not perform as well as expected. This could be because we fully cross-validated these methods when prior studies may not have done so. Hopefully, by releasing our data to the scientific community our results can be confirmed by other laboratories.

We can explain the improved performance of **ETD **over **BKD **on the ChemDB and MUV data. Recall that the Tanimoto metrics used by **ETD **are corrected while the Hamming distance and Simple Matching similarity, used by **BKD**, are not corrected. This would be expected to produce a small performance gain. Experiments not reported here show that, indeed, the performance of the Tanimoto-based methods degrades slightly when the uncorrected version is used in place of the corrected version. This, however, is not enough to fully explain the performance gain; these uncorrected versions still outperform **BKD**. This suggests that part of the performance gain is a direct result of building the method on the Tanimoto similarity rather than the Hamming distance.

Furthermore, we can explain the strong performance of the top methods by noting that most other methods implicitly assume that the chemicals in a given activity class are all similar to one another, that they are all in the same cluster of similar molecules. For example, summing or averaging similarities together assumes that the most likely chemicals are similar to *all *the chemicals in the query and by averaging similarities we are measuring their membership in this 'one-cluster' in a cleaner way. In contrast, for some parameters, the **BKD**, **TPD **and **ETD **a molecule's score can be large if only one query molecule is very similar, regardless of how dissimilar the other query molecules are.

**Figure 8:**
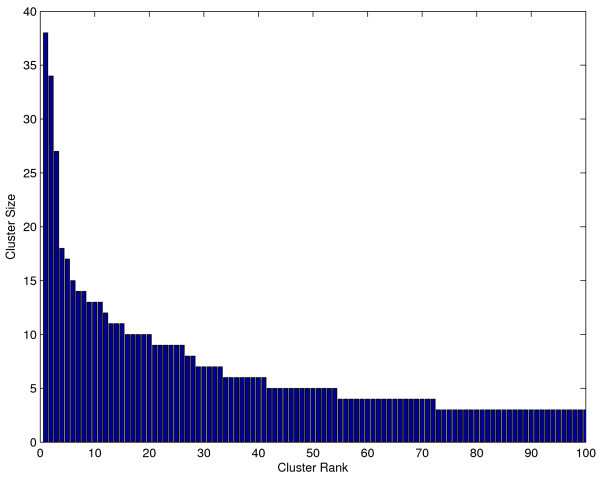
**Size distribution of clusters of the HIV data set**. Clustering the active and moderately-active compounds from the NCI HIV screen using the QT (Quality Threshold) clustering algorithm [28], using the Tanimoto distance and 0.5 as the cluster diameter parameter, produces a few large clusters and many smaller clusters. The size of the one hundred largest clusters found by this method are plotted in decreasing order. These sizes follow a power-law distribution.

We know, in fact, that this 'one-cluster' assumption is not valid on most data sets. For example, clustering the active and moderately-active compounds from the NCI HIV screen using the Quality Threshold clustering algorithm [28], the Tanimoto distance, and 0.5 as the cluster diameter parameter, produces a few large clusters and many smaller clusters (see Figure 8). Most of these clusters may have only a few chemicals in them but some may have many. There are inactive compounds which are close to the aggregate center of the large clusters. This makes biological sense. Each cluster could represent different classes of entirely different molecules. In the case of screening data, different classes could interact with a different protein, different binding sites on the same protein, or, perhaps more importantly, they could represent compounds with different scaffolds interacting with the same protein at the same binding-site. One can imagine inactive compounds which would be ranked inappropriately as actives by 'one-cluster' methods because they have features in common with disparate classes.

It may be more appropriate to first divide up the query into clusters, and then score the database by their distance from the closest cluster. For example, to reformulate the **BAYES **method which typically makes one model of the activity class, we could first segment the query into clusters and model each cluster using a separate Bayesian model. This type of mixture modeling could yield some small performance gains but substantially increases the complexity of the method.

The **MAX-SIM **method has a nice interpretation which does not make the 'one-cluster' assumption. If we assume (1) the probability a given molecule (ℬ) has the same activity (*A*) as a query molecule () is some positive-monotonic function, *f*, of their similarity [29], i.e. *P*(*A*|*S*(, ℬ)) = *f*(*S*(, ℬ)), and (2) this function, *f*, does not vary significantly across chemical space, then sorting chemicals by their maximum similarity to a set of query molecules is a natural way of finding additional molecules with the highest probability of having the same activity.

This interpretation helps us direct future work. For example, by relaxing the second assumption; perhaps the similarity can be tuned by additional indicators, such as the size of the molecule, its solubility, etc., to create a more explicit model of this probability function which would vary appropriately across chemical space. It is also possible that a more rigorous probabilistic framework could further improve retrieval accuracy.

Furthermore, in one of our data sets, the HIV screening data, we only use the 423 confirmed active data points of a 42,682 data point set which also includes 41,175 inactive molecules and 1,081 moderately-active molecules. It should be possible to integrate all this information, to make even better methods for querying databases for molecules with similar activity. Of course, when our query set has been increased to include thousands of molecules of multiple classes, we must design algorithms which not only produce results of biological relevance but also do so efficiently. A robust, efficient algorithm could extrapolate high throughput screening data (which is becoming increasingly available) to help annotate larger databases like PubChem and the ChemDB, and generate hypotheses for additional biological experiments.

Knowing that these are the best multiple-molecule methods helps direct future algorithmic work. The fastest database search methods [30,31] are sub-linear in the size of the chemical database, but remain linear in the number of the query molecules. It should be possible to further decrease the search complexity by using a similar bounding technique to index the query molecules in addition to indexing the database.

## Competing interests

The authors declare that they have no competing interests.

## Authors' contributions

The contributions of SS and RN should be considered equal. SS designed the new methods described in this manuscript and advised RN as he implemented each method, organized the data, and ran the benchmarks. SS and RN wrote the manuscript together. PB provided computational resources in addition to critical advice and editing of the content, structure and organization of this manuscript. All authors read and approved the final manuscript.

## Supplementary Material

Additional file 1 **xvalranks**. The CSV file contains all the ranks of cross-validated active compounds per data set per similarity method. The first entry on each line is the data set, the second entry is the similarity method, and the rest of the entries are the ranks of the actives when cross validated over the entire data set. Click here for file

Additional file 2 **performance**. The CSV file contains all the results of the cross-validated experiments per data set per similarity method. On each line, the first entry is the data set, the second entry is the similarity method, the third, fourth, fifth, and sixth entries are the different performance metrics respectively: AUC, AUAC, F1, and BEDROC Click here for file

Additional file 3 **performance. **The PDF file contains all the results of the cross-validated experiments per data set per similarity method in different tables. The highest performance of each data set is shown in **bold**. The columns, left to right, are: data set, similarity method, AUC, AUAC, F1, and BEDROC. Click here for file
