# Supplementary material for: Large scale study of multiple-molecule queries
Source: J Cheminform. 2009 Jun 4;1:7. doi: 10.1186/1758-2946-1-7 (PMC3225883; doi:10.1186/1758-2946-1-7)
Supplement: Additional file 3 — performance. The PDF file contains all the results of the cross-validated experiments per data set per similarity method in different tables. The highest performance of each data set is shown in bold. The columns, left to right, are: data set, similarity method, AUC, AUAC, F1, and BEDROC. [file 1758-2946-1-7-S3.pdf]

The tables below list the complete results data set by data set of all the methods discussed in the manuscript. The highest performance per data set is shown in **bold**.

Dataset	Method	AUC	AUAC	F1	BEDROC
muv-gpcr-2	MAX-SIM	0.7216	0.7205	0.0667	0.3170
	MIN-SIM	0.4794	0.4784	0.0000	0.0394
	SUM-SIM	0.4902	0.4892	0.0000	0.0347
	NUMDEN-SIM	0.5191	0.5181	0.0000	0.0640
	MIN-RANK	0.7118	0.7107	**0.1333**	**0.3307**
	SUM-RANK	0.4556	0.4546	0.0000	0.0485
	MAX-RANK	0.4289	0.4279	0.0000	0.0166
	BAYES	0.5903	0.5893	0.0000	0.0909
	BKD	0.7417	0.7407	0.0333	0.2120
	ETD	0.7554	0.7544	0.0000	0.2439
	TPD	**0.7768**	**0.7758**	0.1000	0.3093
	SUM-EH	0.6921	0.6910	0.0667	0.1790
	SUM-ET	0.7220	0.7210	0.0667	0.3182
	SUM-TP	0.7216	0.7206	0.0667	0.3189
wom-estrogen	MAX-SIM	0.9936	0.9934	0.5000	0.9156
	MIN-SIM	0.6578	0.6576	0.0000	0.1175
	SUM-SIM	0.9622	0.9620	0.1250	0.8717
	NUMDEN-SIM	0.9604	0.9602	0.1562	0.8706
	MIN-RANK	0.9979	0.9977	0.4844	0.9622
	SUM-RANK	0.9398	0.9396	0.0625	0.6977
	MAX-RANK	0.6027	0.6025	0.0000	0.0985
	BAYES	0.9655	0.9653	0.0781	0.6736
	BKD	0.9943	0.9941	0.4375	0.9310
	ETD	**0.9986**	**0.9984**	0.5000	**0.9698**
	TPD	0.9971	0.9969	0.3594	0.9466
	SUM-EH	0.9614	0.9612	0.0938	0.6881
	SUM-ET	0.9952	0.9950	0.5000	0.9380
	SUM-TP	0.9958	0.9956	**0.5156**	0.9458
suth-steroid	MAX-SIM	0.9998	0.9997	0.4286	0.9936
	MIN-SIM	0.9869	0.9868	0.1429	0.8616
	SUM-SIM	0.9995	0.9994	0.4286	0.9878
	NUMDEN-SIM	0.9994	0.9993	0.4643	0.9875
	MIN-RANK	0.9997	0.9997	0.3929	0.9932
	SUM-RANK	0.9969	0.9968	0.1786	0.9418
	MAX-RANK	0.9309	0.9308	0.0357	0.6701
	BAYES	0.9996	0.9995	**0.6429**	0.9901
	BKD	0.9995	0.9994	0.4286	0.9882
	ETD	0.9998	0.9997	0.3929	0.9936
	TPD	0.9995	0.9994	0.3214	0.9893
	SUM-EH	0.9997	0.9996	0.5000	0.9922
	SUM-ET	0.9998	0.9997	0.4286	0.9937
	SUM-TP	**0.9998**	**0.9997**	0.4286	**0.9938**

Dataset	Method	AUC	AUAC	F1	BEDROC
suth-dhfr	MAX-SIM	0.9998	0.9978	**0.9584**	0.9576
	MIN-SIM	0.7295	0.7274	0.0596	0.2646
	SUM-SIM	0.9844	0.9823	0.8864	0.9156
	NUMDEN-SIM	0.9829	0.9809	0.8837	0.9130
	MIN-RANK	0.9998	0.9978	0.9432	0.9570
	SUM-RANK	0.9798	0.9777	0.8061	0.9042
	MAX-RANK	0.7253	0.7232	0.0208	0.1884
	BAYES	0.9830	0.9809	0.6385	0.8454
	BKD	0.9992	0.9971	0.9238	0.9504
	ETD	**1.0000**	**0.9979**	0.9557	**0.9588**
	TPD	0.9999	0.9979	0.9446	0.9586
	SUM-EH	0.9898	0.9878	0.7327	0.8878
	SUM-ET	0.9999	0.9978	0.9571	0.9582
	SUM-TP	0.9999	0.9978	0.9557	0.9583
suth-benzodiazepine	MAX-SIM	0.9991	0.9980	0.8465	0.9649
	MIN-SIM	0.5891	0.5879	0.0074	0.1221
	SUM-SIM	0.8972	0.8961	0.4703	0.6758
	NUMDEN-SIM	0.8607	0.8596	0.3020	0.5515
	MIN-RANK	**0.9991**	**0.9980**	0.7574	0.9619
	SUM-RANK	0.6505	0.6493	0.0025	0.0862
	MAX-RANK	0.4925	0.4914	0.0000	0.0478
	BAYES	0.9401	0.9389	0.1510	0.5553
	BKD	0.9934	0.9922	0.7351	0.9109
	ETD	0.9970	0.9959	**0.8589**	**0.9664**
	TPD	0.9975	0.9964	0.7871	0.9601
	SUM-EH	0.9633	0.9622	0.4777	0.7501
	SUM-ET	0.9991	0.9979	0.8490	0.9648
	SUM-TP	0.9991	0.9979	0.8490	0.9649
wom-egfr	MAX-SIM	0.9454	0.9451	0.3784	0.7596
	MIN-SIM	0.5265	0.5263	0.0135	0.1049
	SUM-SIM	0.8803	0.8801	0.1892	0.5811
	NUMDEN-SIM	0.8741	0.8738	0.1486	0.5542
	MIN-RANK	0.9636	0.9634	0.3649	0.8246
	SUM-RANK	0.7301	0.7299	0.0405	0.2120
	MAX-RANK	0.4456	0.4454	0.0000	0.1141
	BAYES	0.8724	0.8722	0.0135	0.4611
	BKD	0.9671	0.9669	**0.3919**	0.7924
	ETD	**0.9725**	**0.9723**	**0.3919**	**0.8273**
	TPD	0.9719	0.9717	0.2703	0.7949
	SUM-EH	0.8899	0.8897	0.1351	0.5567
	SUM-ET	0.9472	0.9470	**0.3919**	0.7683
	SUM-TP	0.9474	0.9472	0.3649	0.7729

Dataset	Method	AUC	AUAC	F1	BEDROC
muv-kinase-3	MAX-SIM	0.7520	0.7510	0.1667	0.2649
	MIN-SIM	0.6691	0.6681	0.0000	0.0801
	SUM-SIM	0.6376	0.6366	0.0000	0.1077
	NUMDEN-SIM	0.6572	0.6562	0.0000	0.1370
	MIN-RANK	0.8117	0.8107	**0.2000**	0.3516
	SUM-RANK	0.6393	0.6383	0.0000	0.1112
	MAX-RANK	0.5605	0.5595	0.0000	0.1242
	BAYES	0.6178	0.6167	0.0000	0.1328
	BKD	**0.8889**	**0.8879**	0.0000	0.3255
	ETD	0.8864	0.8854	0.0000	0.3532
	TPD	0.8340	0.8329	0.1667	**0.3891**
	SUM-EH	0.6526	0.6516	0.0667	0.2098
	SUM-ET	0.7574	0.7564	0.1667	0.2680
	SUM-TP	0.7597	0.7587	0.1667	0.2675
wom-cdk2	MAX-SIM	0.9618	0.9614	0.6974	0.8767
	MIN-SIM	0.6918	0.6914	0.0000	0.0972
	SUM-SIM	0.8241	0.8237	0.2105	0.3982
	NUMDEN-SIM	0.8034	0.8030	0.1447	0.3690
	MIN-RANK	0.9770	0.9765	0.5329	0.8926
	SUM-RANK	0.7102	0.7097	0.0132	0.1824
	MAX-RANK	0.5999	0.5995	0.0000	0.1013
	BAYES	0.8204	0.8200	0.0132	0.2282
	BKD	0.9825	0.9821	**0.7632**	0.9002
	ETD	**0.9852**	**0.9847**	0.7237	**0.9181**
	TPD	0.9839	0.9834	0.6711	0.9114
	SUM-EH	0.8923	0.8918	0.1579	0.4697
	SUM-ET	0.9618	0.9614	0.7039	0.8781
	SUM-TP	0.9623	0.9619	0.7039	0.8784
stahl-p38-map-kinase	MAX-SIM	0.9916	0.9916	0.4167	0.9473
	MIN-SIM	0.9917	0.9916	0.0833	0.8809
	SUM-SIM	0.9959	0.9958	0.3750	0.9548
	NUMDEN-SIM	**0.9969**	**0.9969**	**0.5417**	0.9615
	MIN-RANK	0.9964	0.9963	0.3333	0.9602
	SUM-RANK	0.9962	0.9961	0.2917	0.9374
	MAX-RANK	0.9506	0.9505	0.0000	0.5783
	BAYES	0.9834	0.9833	0.1667	0.8542
	BKD	0.9916	0.9915	0.4583	0.9411
	ETD	0.9924	0.9923	0.4583	0.9479
	TPD	0.9900	0.9900	0.4167	0.9384
	SUM-EH	0.9831	0.9830	0.2083	0.8518
	SUM-ET	0.9969	0.9968	0.4583	**0.9619**
	SUM-TP	0.9963	0.9963	0.4583	0.9606

Dataset	Method	AUC	AUAC	F1	BEDROC
wom-pde5	MAX-SIM	0.9895	0.9893	0.6364	0.9276
	MIN-SIM	0.8043	0.8040	0.0114	0.2799
	SUM-SIM	0.9747	0.9745	0.1818	0.7414
	NUMDEN-SIM	0.9695	0.9692	0.1250	0.6870
	MIN-RANK	**0.9939**	**0.9936**	0.5568	0.9417
	SUM-RANK	0.9058	0.9055	0.0000	0.3674
	MAX-RANK	0.7183	0.7180	0.0000	0.1857
	BAYES	0.9379	0.9376	0.0114	0.5397
	BKD	0.9874	0.9871	**0.7500**	0.9248
	ETD	0.9875	0.9873	0.7386	**0.9458**
	TPD	0.9888	0.9886	0.6818	0.9390
	SUM-EH	0.9669	0.9667	0.2500	0.7528
	SUM-ET	0.9929	0.9926	0.7159	0.9353
	SUM-TP	0.9927	0.9925	0.7273	0.9352
wom-d2	MAX-SIM	0.9901	0.9892	0.6677	0.9075
	MIN-SIM	0.8404	0.8394	0.0629	0.3921
	SUM-SIM	0.9546	0.9537	0.2754	0.7420
	NUMDEN-SIM	0.9506	0.9496	0.2665	0.7184
	MIN-RANK	0.9959	0.9950	0.5808	0.9344
	SUM-RANK	0.9314	0.9304	0.1078	0.5796
	MAX-RANK	0.7772	0.7763	0.0479	0.2630
	BAYES	0.9077	0.9067	0.0150	0.3799
	BKD	0.9963	0.9954	0.7036	0.9293
	ETD	0.9951	0.9941	**0.7246**	**0.9431**
	TPD	**0.9964**	**0.9955**	0.7126	0.9387
	SUM-EH	0.9151	0.9141	0.0629	0.4551
	SUM-ET	0.9908	0.9898	0.6766	0.9115
	SUM-TP	0.9907	0.9897	0.6707	0.9116
muv-ppi-3	MAX-SIM	0.6714	0.6704	0.0667	0.1439
	MIN-SIM	0.5118	0.5107	0.0000	0.0616
	SUM-SIM	0.6662	0.6652	0.0000	0.0830
	NUMDEN-SIM	0.7053	0.7043	0.0000	0.1308
	MIN-RANK	0.6592	0.6582	0.0667	0.1818
	SUM-RANK	0.6858	0.6847	0.0000	0.1325
	MAX-RANK	0.6426	0.6416	0.0000	0.1344
	BAYES	0.5750	0.5739	0.0000	0.0244
	BKD	**0.7334**	**0.7324**	**0.1000**	**0.2195**
	ETD	0.7257	0.7246	0.0667	0.1950
	TPD	0.7234	0.7224	**0.1000**	0.2074
	SUM-EH	0.5696	0.5686	0.0000	0.0687
	SUM-ET	0.7200	0.7190	0.0667	0.1546
	SUM-TP	0.7185	0.7175	0.0333	0.1631

Dataset	Method	AUC	AUAC	F1	BEDROC
muv-ppi-1	MAX-SIM	0.6292	0.6282	**0.1333**	0.2098
	MIN-SIM	0.4804	0.4794	0.0000	0.0697
	SUM-SIM	0.4250	0.4239	0.0000	0.0293
	NUMDEN-SIM	0.4563	0.4553	0.0000	0.0390
	MIN-RANK	0.6308	0.6297	0.1000	**0.2237**
	SUM-RANK	0.4331	0.4320	0.0000	0.0456
	MAX-RANK	0.4094	0.4084	0.0000	0.0287
	BAYES	0.5298	0.5288	0.0000	0.0749
	BKD	**0.6828**	**0.6817**	0.0667	0.1810
	ETD	0.6812	0.6802	0.0667	0.1904
	TPD	0.6756	0.6746	**0.1333**	0.2091
	SUM-EH	0.5819	0.5808	0.1000	0.1965
	SUM-ET	0.6271	0.6261	**0.1333**	0.2108
	SUM-TP	0.6292	0.6281	**0.1333**	0.2106
muv-protease-1	MAX-SIM	0.9369	0.9358	**0.4000**	0.6860
	MIN-SIM	0.6445	0.6435	0.0000	0.1093
	SUM-SIM	0.8073	0.8063	0.1667	0.4223
	NUMDEN-SIM	0.8041	0.8030	0.2000	0.4004
	MIN-RANK	0.9692	0.9681	0.2333	0.7275
	SUM-RANK	0.6577	0.6567	0.0000	0.0502
	MAX-RANK	0.5769	0.5758	0.0000	0.0491
	BAYES	0.8530	0.8519	0.0333	0.4286
	BKD	**0.9761**	**0.9751**	**0.4000**	**0.7455**
	ETD	0.9697	0.9687	**0.4000**	0.7164
	TPD	0.9685	0.9674	0.3000	0.7217
	SUM-EH	0.9089	0.9079	0.2667	0.6478
	SUM-ET	0.9381	0.9371	**0.4000**	0.6850
	SUM-TP	0.9379	0.9369	**0.4000**	0.6853
wom-hiv1rt	MAX-SIM	0.9427	0.9424	0.6667	0.8520
	MIN-SIM	0.5554	0.5551	0.0000	0.0846
	SUM-SIM	0.8413	0.8410	0.1515	0.4340
	NUMDEN-SIM	0.8328	0.8325	0.0909	0.3952
	MIN-RANK	0.9439	0.9436	0.5455	0.8537
	SUM-RANK	0.7609	0.7606	0.0000	0.1149
	MAX-RANK	0.5843	0.5840	0.0000	0.0569
	BAYES	0.8409	0.8406	0.0000	0.3240
	BKD	0.9509	0.9506	0.5758	0.7821
	ETD	0.9590	0.9587	**0.7374**	**0.8672**
	TPD	**0.9628**	**0.9625**	0.6869	0.8614
	SUM-EH	0.8876	0.8873	0.1717	0.5091
	SUM-ET	0.9454	0.9452	0.7071	0.8453
	SUM-TP	0.9446	0.9443	0.6869	0.8389

Dataset	Method	AUC	AUAC	F1	BEDROC
stahl-thrombin	MAX-SIM	0.9831	0.9829	0.6418	0.9466
	MIN-SIM	0.7935	0.7933	0.0000	0.2013
	SUM-SIM	0.9466	0.9464	0.5970	0.8271
	NUMDEN-SIM	0.9477	0.9475	0.5970	0.8332
	MIN-RANK	0.9853	0.9851	0.5075	0.9563
	SUM-RANK	0.9310	0.9308	0.1343	0.7257
	MAX-RANK	0.6968	0.6966	0.0000	0.1145
	BAYES	0.8996	0.8994	0.3881	0.6124
	BKD	0.9908	0.9906	**0.7164**	0.9201
	ETD	**0.9965**	**0.9963**	0.6866	**0.9585**
	TPD	0.9958	0.9956	0.6716	0.9484
	SUM-EH	0.8823	0.8821	0.5672	0.6441
	SUM-ET	0.9823	0.9821	0.6418	0.9484
	SUM-TP	0.9830	0.9828	0.6418	0.9478
muv-gpcr-1	MAX-SIM	0.6248	0.6237	0.0000	0.2719
	MIN-SIM	0.5258	0.5248	0.0000	0.0359
	SUM-SIM	0.5368	0.5357	0.0000	0.0396
	NUMDEN-SIM	0.5693	0.5683	0.0000	0.0504
	MIN-RANK	0.6239	0.6229	**0.0667**	0.2641
	SUM-RANK	0.5041	0.5031	0.0000	0.0222
	MAX-RANK	0.5022	0.5011	0.0000	0.0111
	BAYES	0.5369	0.5359	0.0000	0.0969
	BKD	0.6844	0.6834	0.0000	0.2709
	ETD	**0.6920**	**0.6910**	0.0000	**0.2995**
	TPD	0.6792	0.6782	0.0000	0.2735
	SUM-EH	0.5840	0.5830	0.0000	0.0645
	SUM-ET	0.6347	0.6337	0.0000	0.2754
	SUM-TP	0.6337	0.6327	0.0000	0.2728
wom-alr2	MAX-SIM	0.9837	0.9836	0.4524	0.9138
	MIN-SIM	0.5353	0.5352	0.0000	0.0633
	SUM-SIM	0.9334	0.9333	0.2381	0.7361
	NUMDEN-SIM	0.9098	0.9097	0.1905	0.6669
	MIN-RANK	**0.9935**	**0.9934**	**0.5952**	**0.9464**
	SUM-RANK	0.7652	0.7651	0.0000	0.1866
	MAX-RANK	0.3966	0.3964	0.0000	0.0423
	BAYES	0.9778	0.9777	0.0000	0.7290
	BKD	0.9842	0.9841	0.5714	0.8981
	ETD	0.9837	0.9836	**0.5952**	0.9254
	TPD	0.9839	0.9837	0.5000	0.9119
	SUM-EH	0.9837	0.9835	0.2381	0.8329
	SUM-ET	0.9859	0.9858	0.5238	0.9156
	SUM-TP	0.9854	0.9853	0.5714	0.9177

Dataset	Method	AUC	AUAC	F1	BEDROC
muv-protease-2	MAX-SIM	0.9026	0.9015	**0.4000**	0.6207
	MIN-SIM	0.6828	0.6817	0.0333	0.1313
	SUM-SIM	0.9012	0.9001	0.0333	0.4126
	NUMDEN-SIM	0.9145	0.9135	0.0333	0.4494
	MIN-RANK	0.9258	0.9247	**0.4000**	**0.6942**
	SUM-RANK	0.8430	0.8419	0.0000	0.2309
	MAX-RANK	0.5867	0.5856	0.0000	0.0428
	BAYES	0.9235	0.9225	0.0333	0.5669
	BKD	0.9165	0.9155	0.2667	0.6151
	ETD	0.9243	0.9233	0.2667	0.6651
	TPD	0.9197	0.9187	0.2667	0.6174
	SUM-EH	**0.9408**	**0.9397**	0.2000	0.6004
	SUM-ET	0.9366	0.9355	**0.4000**	0.6528
	SUM-TP	0.9358	0.9347	0.3667	0.6534
muv-ppi-2	MAX-SIM	0.6939	0.6929	0.0000	0.2801
	MIN-SIM	0.4659	0.4649	0.0000	0.0612
	SUM-SIM	0.5523	0.5512	0.0000	0.0421
	NUMDEN-SIM	0.5973	0.5963	0.0000	0.0701
	MIN-RANK	0.6963	0.6954	**0.0333**	**0.2874**
	SUM-RANK	0.5598	0.5588	0.0000	0.0408
	MAX-RANK	0.5624	0.5613	0.0000	0.0620
	BAYES	0.6125	0.6114	0.0000	0.0646
	BKD	0.7387	0.7377	0.0000	0.2424
	ETD	**0.7627**	**0.7617**	0.0000	0.2815
	TPD	0.7521	0.7511	0.0000	0.2230
	SUM-EH	0.6004	0.5994	0.0000	0.0954
	SUM-ET	0.6933	0.6923	0.0000	0.2812
	SUM-TP	0.6940	0.6930	0.0000	0.2814
wom-impdh	MAX-SIM	0.9544	0.9543	0.6327	0.8485
	MIN-SIM	0.7022	0.7021	0.0000	0.1480
	SUM-SIM	0.9275	0.9273	**0.7347**	0.8057
	NUMDEN-SIM	0.9276	0.9275	**0.7347**	0.7997
	MIN-RANK	0.9679	0.9677	0.5510	0.8827
	SUM-RANK	0.9136	0.9135	0.0816	0.6674
	MAX-RANK	0.5467	0.5466	0.0000	0.1216
	BAYES	0.9438	0.9437	0.2449	0.7956
	BKD	0.9638	0.9637	**0.7347**	**0.8887**
	ETD	0.9710	0.9708	0.6735	0.8815
	TPD	**0.9737**	**0.9736**	0.5102	0.8777
	SUM-EH	0.9463	0.9461	0.5918	0.8598
	SUM-ET	0.9540	0.9539	0.6327	0.8494
	SUM-TP	0.9542	0.9541	0.6327	0.8491

Dataset	Method	AUC	AUAC	F1	BEDROC
wom-fxa	MAX-SIM	0.9985	0.9982	0.6449	0.9679
	MIN-SIM	0.8209	0.8205	0.1869	0.4273
	SUM-SIM	0.9669	0.9666	0.6542	0.7972
	NUMDEN-SIM	0.9635	0.9632	0.6542	0.7843
	MIN-RANK	**0.9994**	**0.9991**	0.8131	**0.9855**
	SUM-RANK	0.9226	0.9223	0.3458	0.6183
	MAX-RANK	0.7677	0.7674	0.0280	0.2660
	BAYES	0.8879	0.8876	0.1121	0.4601
	BKD	0.9945	0.9942	**0.9159**	0.9747
	ETD	0.9939	0.9936	0.8505	0.9806
	TPD	0.9961	0.9958	0.8411	0.9779
	SUM-EH	0.9514	0.9511	0.4579	0.7709
	SUM-ET	0.9992	0.9989	0.6636	0.9791
	SUM-TP	0.9993	0.9990	0.6916	0.9808
muv-chaperone	MAX-SIM	0.6406	0.6396	0.0667	0.2001
	MIN-SIM	0.5255	0.5244	0.0000	0.0461
	SUM-SIM	0.5732	0.5722	0.0000	0.0671
	NUMDEN-SIM	0.6074	0.6063	0.0000	0.0925
	MIN-RANK	0.6830	0.6819	0.1000	0.3008
	SUM-RANK	0.5619	0.5608	0.0000	0.0550
	MAX-RANK	0.4234	0.4224	0.0000	0.0117
	BAYES	0.5962	0.5951	0.0000	0.1401
	BKD	**0.8129**	**0.8119**	0.1667	**0.4210**
	ETD	0.8096	0.8085	**0.2000**	0.4077
	TPD	0.7607	0.7597	0.1333	0.3999
	SUM-EH	0.6518	0.6507	0.0000	0.1816
	SUM-ET	0.6405	0.6395	0.0667	0.2015
	SUM-TP	0.6410	0.6399	0.0667	0.2016
muv-nr-1	MAX-SIM	0.7294	0.7283	**0.2000**	0.2984
	MIN-SIM	0.5245	0.5235	0.0000	0.0343
	SUM-SIM	0.5949	0.5938	0.0000	0.0407
	NUMDEN-SIM	0.6198	0.6188	0.0000	0.0638
	MIN-RANK	0.6876	0.6866	0.1333	**0.3257**
	SUM-RANK	0.5636	0.5626	0.0000	0.0872
	MAX-RANK	0.5203	0.5192	0.0667	0.0835
	BAYES	0.5781	0.5770	0.0000	0.0854
	BKD	0.7329	0.7319	0.1000	0.2489
	ETD	0.7293	0.7282	0.0667	0.2325
	TPD	0.7102	0.7092	0.1000	0.2662
	SUM-EH	0.6216	0.6205	0.0000	0.0573
	SUM-ET	**0.7488**	**0.7478**	**0.2000**	0.3019
	SUM-TP	0.7474	0.7463	**0.2000**	0.3041

Dataset	Method	AUC	AUAC	F1	BEDROC
nci-hiv	MAX-SIM	0.9320	0.9308	0.7855	0.8610
	MIN-SIM	0.4715	0.4703	0.0000	0.0295
	SUM-SIM	0.7156	0.7144	0.2627	0.4018
	NUMDEN-SIM	0.6738	0.6726	0.1735	0.3051
	MIN-RANK	0.9583	0.9571	0.6482	0.8726
	SUM-RANK	0.4128	0.4116	0.0000	0.0103
	MAX-RANK	0.3182	0.3170	0.0000	0.0096
	BAYES	0.6125	0.6113	0.0024	0.0546
	BKD	0.9758	0.9746	0.7566	0.8708
	ETD	**0.9804**	**0.9792**	**0.8120**	**0.8894**
	TPD	0.9789	0.9777	0.7518	0.8865
	SUM-EH	0.8547	0.8535	0.2169	0.5143
	SUM-ET	0.9316	0.9304	0.7880	0.8612
	SUM-TP	0.9319	0.9307	0.7880	0.8612
wom-ppar-gamma	MAX-SIM	0.9717	0.9717	0.5926	0.9208
	MIN-SIM	0.9311	0.9311	0.1111	0.6961
	SUM-SIM	0.9787	0.9786	0.2963	0.9120
	NUMDEN-SIM	0.9817	0.9816	0.3333	0.9157
	MIN-RANK	0.9856	0.9855	0.6296	0.9266
	SUM-RANK	0.9759	0.9758	0.2593	0.8377
	MAX-RANK	0.9084	0.9083	0.1111	0.6372
	BAYES	0.9389	0.9388	0.0000	0.6593
	BKD	0.9984	0.9983	**0.7037**	**0.9734**
	ETD	**0.9984**	**0.9984**	0.6667	0.9734
	TPD	0.9959	0.9958	0.6296	0.9506
	SUM-EH	0.9736	0.9735	0.4815	0.8656
	SUM-ET	0.9829	0.9828	0.6296	0.9239
	SUM-TP	0.9831	0.9831	0.6296	0.9234
wom-p38-map-kinase	MAX-SIM	0.9761	0.9759	0.5593	0.8761
	MIN-SIM	0.6653	0.6651	0.0000	0.1046
	SUM-SIM	0.9467	0.9465	0.2034	0.6538
	NUMDEN-SIM	0.9400	0.9399	0.1695	0.6152
	MIN-RANK	0.9925	0.9923	0.5254	0.9294
	SUM-RANK	0.8469	0.8468	0.0000	0.2195
	MAX-RANK	0.5093	0.5091	0.0000	0.1104
	BAYES	0.9335	0.9333	0.0000	0.5511
	BKD	0.9855	0.9853	0.5593	0.9024
	ETD	**0.9965**	**0.9963**	**0.5932**	**0.9528**
	TPD	0.9934	0.9933	0.5254	0.9273
	SUM-EH	0.9481	0.9479	0.2542	0.7151
	SUM-ET	0.9892	0.9891	0.5593	0.9050
	SUM-TP	0.9887	0.9885	0.5254	0.9004

Dataset	Method	AUC	AUAC	F1	BEDROC
wom-androgen	MAX-SIM	0.9053	0.9052	0.2778	0.7304
	MIN-SIM	0.4414	0.4413	0.0000	0.0394
	SUM-SIM	0.6662	0.6661	0.0833	0.3334
	NUMDEN-SIM	0.6597	0.6595	0.0278	0.3264
	MIN-RANK	0.9183	0.9182	0.2778	0.7467
	SUM-RANK	0.4992	0.4991	0.0000	0.0195
	MAX-RANK	0.4080	0.4079	0.0000	0.0097
	BAYES	0.8522	0.8521	0.0000	0.4082
	BKD	0.9099	0.9098	**0.3056**	0.5968
	ETD	**0.9591**	**0.9590**	**0.3056**	**0.7647**
	TPD	0.9538	0.9537	0.2500	0.6923
	SUM-EH	0.8437	0.8436	0.0278	0.4784
	SUM-ET	0.9035	0.9034	0.2778	0.7306
	SUM-TP	0.9045	0.9044	0.2778	0.7311
muv-nr-2	MAX-SIM	0.4977	0.4967	**0.0000**	0.0425
	MIN-SIM	0.4756	0.4745	**0.0000**	0.0367
	SUM-SIM	0.4595	0.4584	**0.0000**	0.0126
	NUMDEN-SIM	0.5147	0.5136	**0.0000**	0.0313
	MIN-RANK	0.4891	0.4880	**0.0000**	0.0770
	SUM-RANK	0.5142	0.5132	**0.0000**	0.0381
	MAX-RANK	0.4931	0.4920	**0.0000**	0.0651
	BAYES	0.5639	0.5629	**0.0000**	0.0468
	BKD	0.5962	0.5952	**0.0000**	0.0697
	ETD	0.5918	0.5908	**0.0000**	0.0718
	TPD	**0.5981**	**0.5970**	**0.0000**	**0.0912**
	SUM-EH	0.5437	0.5427	**0.0000**	0.0111
	SUM-ET	0.5238	0.5228	**0.0000**	0.0362
	SUM-TP	0.5145	0.5135	**0.0000**	0.0317
muv-protease-3	MAX-SIM	0.8583	0.8572	0.3333	0.5259
	MIN-SIM	0.6609	0.6598	0.0000	0.1020
	SUM-SIM	0.8043	0.8033	0.3333	0.4387
	NUMDEN-SIM	0.8264	0.8254	0.3000	0.4616
	MIN-RANK	0.8907	0.8897	0.1667	0.5313
	SUM-RANK	0.7971	0.7960	0.0333	0.3317
	MAX-RANK	0.6398	0.6388	0.0000	0.0582
	BAYES	0.8035	0.8025	0.1000	0.4033
	BKD	0.8919	0.8909	**0.3667**	**0.6154**
	ETD	**0.9033**	**0.9023**	**0.3667**	0.5935
	TPD	0.9033	0.9023	0.3333	0.5969
	SUM-EH	0.8138	0.8128	0.3333	0.4430
	SUM-ET	0.8727	0.8716	**0.3667**	0.5289
	SUM-TP	0.8710	0.8700	**0.3667**	0.5250

Dataset	Method	AUC	AUAC	F1	BEDROC
muv-kinase-1	MAX-SIM	0.7535	0.7524	0.1333	0.3785
	MIN-SIM	0.5104	0.5093	0.0333	0.0948
	SUM-SIM	0.8112	0.8102	0.1333	0.4558
	NUMDEN-SIM	0.8336	0.8326	0.1000	0.4969
	MIN-RANK	0.7535	0.7525	0.1000	0.3363
	SUM-RANK	0.7899	0.7888	0.0000	0.2521
	MAX-RANK	0.4922	0.4911	0.0000	0.1367
	BAYES	0.7650	0.7640	0.0667	0.3377
	BKD	**0.8566**	**0.8555**	0.1333	0.5099
	ETD	0.8535	0.8525	0.1667	**0.5146**
	TPD	0.8379	0.8368	**0.2000**	0.4741
	SUM-EH	0.8019	0.8008	0.1333	0.4160
	SUM-ET	0.8353	0.8342	0.1333	0.5105
	SUM-TP	0.8076	0.8065	0.1333	0.4889
stahl-neuraminidase	MAX-SIM	0.9999	0.9999	**0.8235**	0.9977
	MIN-SIM	0.9992	0.9991	0.2353	0.9827
	SUM-SIM	0.9993	0.9992	0.4118	0.9852
	NUMDEN-SIM	0.9997	0.9996	0.5882	0.9925
	MIN-RANK	**1.0000**	**0.9999**	0.5882	**0.9985**
	SUM-RANK	0.9950	0.9949	0.1176	0.9213
	MAX-RANK	0.9947	0.9947	0.0588	0.9138
	BAYES	0.9966	0.9966	0.1765	0.9446
	BKD	0.9999	0.9999	0.6471	0.9980
	ETD	1.0000	0.9999	0.7647	0.9982
	TPD	0.9994	0.9994	0.3529	0.9882
	SUM-EH	0.9991	0.9991	0.3529	0.9817
	SUM-ET	0.9999	0.9999	0.7647	0.9977
	SUM-TP	0.9999	0.9999	0.7647	0.9977
suth-estrogen	MAX-SIM	0.9995	0.9985	0.9501	0.9766
	MIN-SIM	0.8476	0.8465	0.0443	0.2327
	SUM-SIM	0.9952	0.9942	0.8255	0.9609
	NUMDEN-SIM	0.9949	0.9939	0.8172	0.9567
	MIN-RANK	0.9997	0.9987	0.8837	0.9754
	SUM-RANK	0.9910	0.9900	0.6676	0.9296
	MAX-RANK	0.5314	0.5304	0.0277	0.0629
	BAYES	0.9961	0.9950	0.6454	0.9427
	BKD	0.9991	0.9980	0.8947	0.9724
	ETD	**0.9999**	**0.9989**	0.9474	**0.9787**
	TPD	0.9999	0.9989	0.9169	0.9783
	SUM-EH	0.9963	0.9952	0.7562	0.9509
	SUM-ET	0.9995	0.9985	**0.9557**	0.9765
	SUM-TP	0.9995	0.9985	**0.9557**	0.9765

Dataset	Method	AUC	AUAC	F1	BEDROC
stahl-cox2	MAX-SIM	0.9814	0.9810	0.7120	0.9324
	MIN-SIM	0.5328	0.5325	0.0000	0.0516
	SUM-SIM	0.8971	0.8967	0.5200	0.6929
	NUMDEN-SIM	0.8918	0.8915	0.5280	0.6875
	MIN-RANK	0.9853	0.9850	0.6080	0.9406
	SUM-RANK	0.8569	0.8565	0.0720	0.5521
	MAX-RANK	0.5073	0.5069	0.0000	0.0291
	BAYES	0.9169	0.9165	0.0720	0.5644
	BKD	0.9793	0.9789	0.6640	0.8937
	ETD	0.9893	0.9890	**0.7360**	**0.9465**
	TPD	**0.9899**	**0.9895**	0.7120	0.9424
	SUM-EH	0.9228	0.9224	0.3520	0.6292
	SUM-ET	0.9810	0.9806	0.7120	0.9324
	SUM-TP	0.9814	0.9810	0.7120	0.9325
muv-rtk	MAX-SIM	0.6975	0.6965	0.0667	0.2809
	MIN-SIM	0.4617	0.4607	0.0333	0.0701
	SUM-SIM	0.5356	0.5346	0.0000	0.0900
	NUMDEN-SIM	0.5738	0.5727	0.0000	0.1175
	MIN-RANK	0.7318	0.7307	0.0667	0.2504
	SUM-RANK	0.5154	0.5143	0.0333	0.0448
	MAX-RANK	0.4604	0.4594	0.0000	0.0512
	BAYES	0.5468	0.5458	0.0000	0.0929
	BKD	0.8100	0.8089	**0.1000**	**0.3401**
	ETD	**0.8109**	**0.8099**	0.0667	0.3121
	TPD	0.7927	0.7917	**0.1000**	0.3119
	SUM-EH	0.6223	0.6213	0.0667	0.1536
	SUM-ET	0.6994	0.6984	0.0667	0.2819
	SUM-TP	0.6988	0.6978	0.0667	0.2829
stahl-gelatinase	MAX-SIM	0.9315	0.9314	0.1000	0.7185
	MIN-SIM	0.7238	0.7237	0.0000	0.2371
	SUM-SIM	0.8912	0.8911	0.0250	0.5803
	NUMDEN-SIM	0.8922	0.8920	0.0250	0.5653
	MIN-RANK	0.9533	0.9532	0.2500	0.8332
	SUM-RANK	0.7943	0.7942	0.0000	0.3090
	MAX-RANK	0.5223	0.5222	0.0000	0.1325
	BAYES	0.8291	0.8290	0.0000	0.2314
	BKD	0.9748	0.9747	**0.3000**	**0.8843**
	ETD	**0.9769**	**0.9768**	0.2500	0.8777
	TPD	0.9726	0.9725	0.1250	0.7920
	SUM-EH	0.8390	0.8389	0.1000	0.3765
	SUM-ET	0.9295	0.9294	0.1000	0.7191
	SUM-TP	0.9310	0.9309	0.1000	0.7191

Dataset	Method	AUC	AUAC	F1	BEDROC
muv-gpcr-3	MAX-SIM	0.5457	0.5446	0.0000	0.1325
	MIN-SIM	0.5345	0.5335	**0.0333**	0.0570
	SUM-SIM	0.5584	0.5574	0.0000	0.0949
	NUMDEN-SIM	0.6070	0.6059	0.0000	0.1364
	MIN-RANK	0.5766	0.5756	**0.0333**	0.1332
	SUM-RANK	0.6029	0.6019	**0.0333**	0.0870
	MAX-RANK	0.4695	0.4685	**0.0333**	0.0375
	BAYES	0.5486	0.5476	0.0000	0.0524
	BKD	**0.6359**	**0.6349**	0.0000	0.1962
	ETD	0.6262	0.6252	0.0000	**0.2089**
	TPD	0.6188	0.6178	0.0000	0.1365
	SUM-EH	0.5614	0.5603	0.0000	0.0916
	SUM-ET	0.6117	0.6107	0.0000	0.1478
	SUM-TP	0.5998	0.5988	0.0000	0.1505
wom-cox2	MAX-SIM	0.9402	0.9400	0.4474	0.8394
	MIN-SIM	0.5317	0.5315	0.0000	0.0557
	SUM-SIM	0.8035	0.8033	0.3026	0.4713
	NUMDEN-SIM	0.7985	0.7982	0.2368	0.4632
	MIN-RANK	0.9536	0.9533	0.5263	0.8700
	SUM-RANK	0.7198	0.7196	0.0000	0.1527
	MAX-RANK	0.5480	0.5478	0.0000	0.0489
	BAYES	0.8558	0.8555	0.0000	0.4018
	BKD	0.9362	0.9360	0.4868	0.7685
	ETD	0.9698	0.9695	**0.5789**	**0.8840**
	TPD	**0.9716**	**0.9713**	0.5000	0.8660
	SUM-EH	0.8740	0.8738	0.0789	0.5040
	SUM-ET	0.9398	0.9396	0.4474	0.8399
	SUM-TP	0.9400	0.9397	0.4474	0.8407
muv-rnase	MAX-SIM	0.6955	0.6945	0.0667	0.2443
	MIN-SIM	0.2680	0.2670	0.0000	0.0229
	SUM-SIM	0.4483	0.4473	0.0000	0.0102
	NUMDEN-SIM	0.4655	0.4645	0.0000	0.0195
	MIN-RANK	0.7611	0.7601	**0.1000**	0.3487
	SUM-RANK	0.4291	0.4281	0.0000	0.0191
	MAX-RANK	0.3306	0.3297	0.0000	0.0177
	BAYES	0.5105	0.5094	0.0000	0.0874
	BKD	0.7814	0.7804	0.0667	**0.3516**
	ETD	0.7817	0.7807	0.0667	0.3239
	TPD	**0.7836**	**0.7826**	**0.1000**	0.3371
	SUM-EH	0.6074	0.6064	0.0000	0.1652
	SUM-ET	0.7117	0.7107	0.0333	0.2563
	SUM-TP	0.6946	0.6936	0.0667	0.2460

Dataset	Method	AUC	AUAC	F1	BEDROC
muv-kinase-2	MAX-SIM	0.8183	0.8173	0.2000	0.4529
	MIN-SIM	0.6484	0.6474	0.0000	0.1439
	SUM-SIM	0.7046	0.7036	0.0000	0.2460
	NUMDEN-SIM	0.7011	0.7000	0.0000	0.2709
	MIN-RANK	0.8367	0.8356	0.2000	0.4811
	SUM-RANK	0.6434	0.6424	0.0000	0.1935
	MAX-RANK	0.5734	0.5724	0.0000	0.1166
	BAYES	0.7945	0.7934	0.0667	0.3002
	BKD	0.8661	0.8650	**0.2333**	**0.5146**
	ETD	**0.8736**	**0.8726**	**0.2333**	0.5122
	TPD	0.8700	0.8690	**0.2333**	0.4914
	SUM-EH	0.8136	0.8125	0.1667	0.3692
	SUM-ET	0.8194	0.8183	0.2000	0.4552
	SUM-TP	0.8187	0.8177	0.2000	0.4559
stahl-estrogen	MAX-SIM	0.9984	0.9983	0.5472	0.9720
	MIN-SIM	0.8483	0.8481	0.0000	0.3577
	SUM-SIM	0.9825	0.9823	0.5283	0.9132
	NUMDEN-SIM	0.9814	0.9813	**0.5849**	0.9040
	MIN-RANK	**0.9988**	**0.9986**	0.4906	**0.9777**
	SUM-RANK	0.9588	0.9586	0.0755	0.6700
	MAX-RANK	0.7396	0.7395	0.0189	0.2238
	BAYES	0.9802	0.9800	0.0943	0.8257
	BKD	0.9938	0.9937	**0.5849**	0.9531
	ETD	0.9977	0.9975	0.5283	0.9621
	TPD	0.9957	0.9956	0.4340	0.9387
	SUM-EH	0.9871	0.9870	0.3396	0.8586
	SUM-ET	0.9984	0.9983	0.5472	0.9724
	SUM-TP	0.9985	0.9983	**0.5849**	0.9730
